# Modeling drug response using network-based personalized treatment prediction (NetPTP) with applications to inflammatory bowel disease

**DOI:** 10.1371/journal.pcbi.1008631

**Published:** 2021-02-05

**Authors:** Lichy Han, Zahra N. Sayyid, Russ B. Altman

**Affiliations:** 1 Biomedical Informatics Training Program, Stanford University, Stanford, California, United States of America; 2 Department of Otolaryngology-Head and Neck Surgery, Stanford University School of Medicine, Stanford, California, United States of America; 3 Department of Genetics, Stanford University, Stanford, California, United States of America; 4 Department of Bioengineering, Stanford University, Stanford, California, United States of America; University of California San Diego, UNITED STATES

## Abstract

For many prevalent complex diseases, treatment regimens are frequently ineffective. For example, despite multiple available immunomodulators and immunosuppressants, inflammatory bowel disease (IBD) remains difficult to treat. Heterogeneity in the disease across patients makes it challenging to select the optimal treatment regimens, and some patients do not respond to any of the existing treatment choices. Drug repurposing strategies for IBD have had limited clinical success and have not typically offered individualized patient-level treatment recommendations. In this work, we present NetPTP, a Network-based Personalized Treatment Prediction framework which models measured drug effects from gene expression data and applies them to patient samples to generate personalized ranked treatment lists. To accomplish this, we combine publicly available network, drug target, and drug effect data to generate treatment rankings using patient data. These ranked lists can then be used to prioritize existing treatments and discover new therapies for individual patients. We demonstrate how NetPTP captures and models drug effects, and we apply our framework to individual IBD samples to provide novel insights into IBD treatment.

## Author summary

Offering personalized treatment results is an important tenant of precision medicine, particularly in complex diseases which have high variability in disease manifestation and treatment response. We have developed a novel framework, NetPTP (Network-based Personalized Treatment Prediction), for making personalized drug ranking lists for patient samples. Our method uses networks to model drug effects from gene expression data and applies these captured effects to individual samples to produce tailored drug treatment rankings. We applied NetPTP to inflammatory bowel disease, yielding insights into the treatment of this particular disease. Our method is modular and generalizable, and thus can be applied to other diseases that could benefit from a personalized treatment approach.

## Introduction

Drug development is an expensive and lengthy endeavor, on average costing approximately a billion dollars to successfully bring a drug to market [1]. As such, drug repurposing, also known as drug repositioning, has become an important avenue for discovering existing treatments for new indications, saving time and money in the quest for new therapies. With increasing data available on drugs and diseases, computational approaches for drug repositioning have shown great potential by integrating multiple sources of information to discover novel matchings of drugs and diseases.

Using transcriptomic data, multiple existing computational approaches for drug repurposing are based on constructing representations of diseases and drugs and assessing their similarity. For example, Li and Greene et al used differentially expressed genes to construct and compare disease and drug signatures and van Noort et al applied a similar approach using 500 probe sets in colorectal cancer [2,3]. However, by representing the disease as an aggregate, these methods can be limited in their ability to capture patient and disease heterogeneity. Furthermore, by treating each gene or probe set individually, these methods frequently fail to capture different combinations of perturbations that cause similar disease phenotypes, which contributes to disease heterogeneity. For complex, heterogeneous diseases, there are frequently multiple avenues of treatment targeting different aspects of the disease, and many patients do not respond to the same set of therapies. Such diseases could benefit from a generative method that produces more personalized therapeutic strategies that target an individual’s disease state.

One such condition is inflammatory bowel disease (IBD), which consists of two main subtypes, ulcerative colitis (UC) and Crohn’s disease (CD). Both are chronic inflammatory conditions of the gastrointestinal system which together affect over 1.5 million people in the United States [4]. As a heterogeneous disease, different IBD patients frequently respond to different treatment drugs that target specific pathways unique to the disease pathogenesis seen in that particular patient. As such, there currently exist multiple different treatments for IBD which have different mechanisms of action, such as sulfasalazine, infliximab, azathioprine, and steroids [5]. However, it is frequently unclear which patients would derive the most benefit from each of these classes of drugs. Furthermore, many patients do not respond or develop nonresponse to these therapies, resulting in escalation of their treatment regimens or surgery.

There exist a few previous computational repurposing methods that have been applied to IBD. For example, Dudley et al compared drugged gene expression signatures from the Connectivity Map (CMap) to IBD gene expression data identified topiramate as a potential therapeutic candidate [6]. Another approach overlapped IBD genes implicated in genome wide association studies with known drug targets for IBD [7]. More recently, newer approaches have incorporated gene interactions by examining sets of genes in the same pathway. For example, Grenier et al employed a pathway-based approach using genetic loci from IBD gene wide association studies and pathway set enrichment analysis to identify new candidate drugs [8]. While these methods have yielded some new potential therapies, there is still a great need for identifying responders and for additional therapeutic strategies for nonresponders.

We present Network-based Personalized Treatment Prediction (NetPTP), a novel systems pharmacological approach for modeling drug effects, which incorporates the interactions of genes and proteins with drug targets in order repurpose and prioritize drugs in individual patients. Using publicly available human and mouse gene expression data, we show how our approach can be used to identify drugs based on each patient’s disease profile. We further aggregate these individual results to the disease level to gain new insights into the treatment of CD and UC.

## Methods

### Ethics statement

The experimental portion of this study (protocol ID 32963) was reviewed and approved by the Institutional Animal Care and Use Committee (IACUC) at Stanford University.

### Data preprocessing

#### The connectivity map

We downloaded all instances in CMap as raw CEL files. We first preprocessed the instances using robust multi-array average [9]. Probes were then mapped to genes using the corresponding platform annotation files. For genes corresponding to multiple probes, the expression values were averaged across all probes corresponding to the same gene. We then corrected for batch effects using ComBat [10] with the *sva* package [11], correcting for batch number as given by CMap and for cell line. Only instances which mapped to a drug in DrugBank and had at least one gene target measured were included in downstream analysis.

#### Human IBD disease datasets

We processed four publicly available IBD colonic sample datasets from GEO: GSE16879 [12], GSE9686 [13], GSE10616 [14], and GSE36807 [15] (S1 Table). Each study contains baseline colonic sample prior to treatment of CD and UC patients, and also contains healthy control colonic samples. Of note, GSE16879 consists of patients refractory to corticosteroids and/or immunosuppression, and also contains samples before and after infliximab treatment. We likewise preprocessed these datasets using the same framework as described for the CMap data.

#### Mouse IBD disease datasets

We additionally processed two publicly available IBD mouse model samples: GSE22307 [16] and GSE53835 [17] (S1 Table). GSE22307 used dextran sulfate sodium (DSS) and consisted of a total of 18 mice, where six mice were sacrificed on day 2, 4, and 6 after IBD induction. GSE53835 utilized 2, 4, 6-trinitrobenzenesulfonic acid (TNBS) to induce IBD, and all mice were sacrificed on day 4.

#### Methotrexate response data in rheumatoid arthritis

Aside from IBD, we processed a rheumatoid arthritis dataset, GSE45867 [18], which contains human synovial biopsy samples before and after methotrexate treatment (N = 8) and before and after tocilizumab treatment (N = 12). As tocilizumab is not a drug present CMap, we analyzed only the methotrexate samples, comparing our simulated drugged samples generated from the untreated samples to the measured treated samples.

### Network construction

We next downloaded all drug data available online from DrugBank [19]. We extracted drug targets and converted all gene names to the Entrez gene identifier. Only drugs that were included in CMap and have a gene target that was measured were included in downstream analysis.

We extracted all human pathways from Reactome [20] and connected all pathways into one large network. Only genes that were measured in CMap were included. Undirected edges from Reactome are represented as two directed edges in opposite directions.

**Fig 1:**
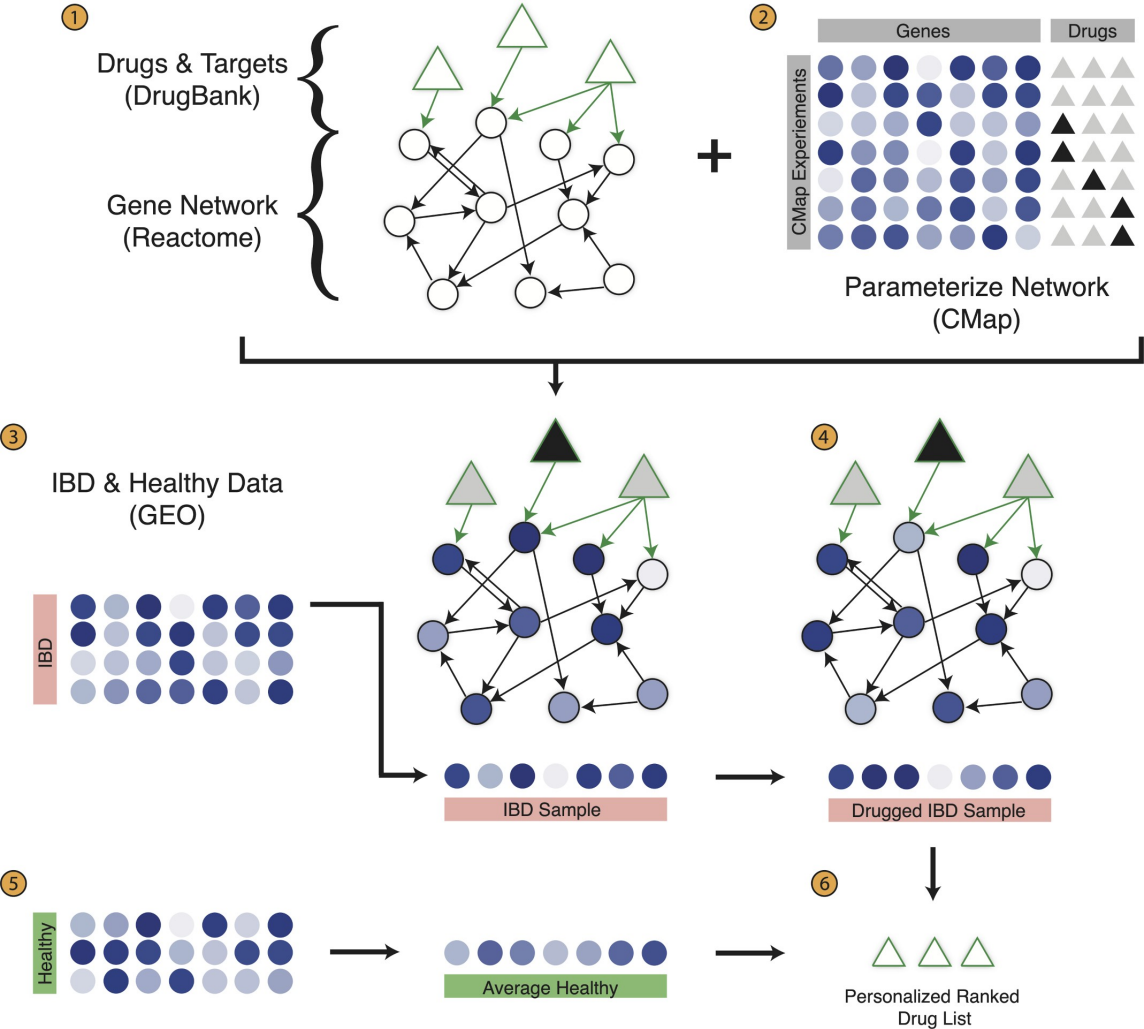
Overview of drug effect modeling. (1) Drugs, drug targets, genes, and gene interactions are curated from DrugBank and Reactome to form a gene-drug network. (2) The expression of each gene is modeled as a linear regression of incoming nodes, where the coefficient parameters are learned from the Connectivity Map. (3) IBD data is curated from the Gene Expression Omnibus (GEO), and for each sample (4) a drugged IBD sample is created using the network. (5) The healthy samples are averaged to create a healthy patient representation, and (6) each drugged IBD sample is compared to the healthy sample using Euclidean distance to create a personalized ranked drug list.

As shown in Fig 1, step 1, each circular node represents a gene, and each directed arrow represents an interaction from Reactome. We next add drug nodes, depicted as triangles, to the overall network or genes (Fig 1, step 1). A drug may have multiple targets, which would be represented as having multiple outgoing edges. Similarly, a gene may be the target of multiple drugs, depicted by multiple incoming edges.

### Drug effect modeling

The network is then parameterized by drugged gene expression data, where each circular gene node is a linear regression of the incoming nodes, and triangular drug nodes are binary variables (Fig 1, step 2). Regularized regression was not used as the median number of input genes for each node, 13, was small compared to the 3,400 training instances, lowering the concern for overfitting. The network of regressions can be intuitively thought of as diffusion of the drug effect through the gene network. Thus, we have now created a model where drugs can be turned “on” or “off”, where the network is capturing the changes induced by these effects.

With the parameterized network modeling drug effects, we then apply this network to IBD disease data in order to discover which drugs may be most effective for each individual patient. For each IBD sample, we take the gene expression values and overlay them onto the nodes of the network. We then turn drugs on or off (Fig 1, step 3), and propagate these effects through the network (Fig 1 step 4). Thus, for each IBD sample, we create a “drugged IBD sample” gene expression sample. Using the healthy data, we average all healthy samples, to create an “average healthy” gene expression sample (Fig 1, step 5). We then compare the average healthy sample to each drugged IBD sample, using Euclidean distance, to create a personalized, ranked drug list (Fig 1, step 6). In essence, we are comparing the effects of different drugs to see which set can bring the original IBD sample closest to the average healthy sample. This way, we discover drugs that not only treat the disease symptoms, but may have fewer side effects than drugs that may have a beneficial effect on the disease but do not result in an expression profile closer to the average healthy sample.

### Curation of IBD drugs in literature

We curated a list of drugs that have been previously studied in the context IBD based on work by Percha, et al. [21,22] Briefly, entities and dependency paths are extracted from over 16 million MEDLINE abstracts. These paths are then clustered using the Ensemble Biclustering for Classification method to produce clusters of entity relationships. Specifically, we used the chemical-disease relationship output, which consists of 6 themes: 1) (T) Treatment/therapy, 2) (C) Inhibits cell growth, 3) (Sa) side effect/adverse event, 4) (Pr) prevents, suppresses, 5) (Pa) alleviates, reduces, 6) (J) role in pathogenesis. For our known drugs, we extracted any drug that had a T, Pr, and/or Pa relationship with CD, UC, and/or IBD.

### Calculating alternative drug-disease score rankings

We compared NetPTP to the drug rankings produced by the method used in Dudley et al [6], as our work uses similar data sources and is also applied to the same disease, IBD. In their method, a drug-disease score (DDS) is derived for each drug based on how anti-correlated the drug signature is as compared to the disease signature. We calculated the DDS as described in Sirota and Dudley et al [23] for all drugs and CD, and all drugs and UC using the samples in GEO9686, GEO36807, and GEO10616. GEO16879 was not included as these patients were refractory to the mainstay treatments for IBD. Following their method, we first used significance analysis of microarrays (SAM) [24] to derive lists of up-regulated and down-regulated genes, comparing diseased samples to the healthy samples in each study. We then calculated the up-regulated and down-regulated enrichment score, and subsequently the drug-disease score (DDS) for each drug-CD and drug-UC pair in each of the three studies.

We then ranked all drugs by their DDS, from most anti-correlated to least anti-correlated. The original method calls for DDS to be set to 0 if the up-regulated enrichment score and down-regulated enrichment score are the same direction, otherwise DDS is set to the difference between the two. This results in a large number of drugs all having a score of 0, and we differentiated drugs with a score of 0 by ranking them by the difference between the up-regulated and down-regulated enrichment scores.

In order to compare the rankings from the two methods, we curated the FDA approved treatments for IBD that are in CMap. These were budesonide, prednisone, prednisolone, methylprednisolone, azathioprine, mercaptopurine, sulfasalazine, mesalazine, and methotrexate. We then compared the rankings of these nine known treatments based on the DDS to our NetPTP rankings across all studies.

### Experimental protocol for drug evaluation in TNBS mice

We conducted a pilot experimental study to assess the top drug prediction in TNBS mice, amrinone. The study was reviewed and approved by the Institutional Animal Care and Use Committee (IACUC) at Stanford University. We purchased twenty 6-week-old C57BL/6 mice from The Jackson Laboratory (Bar Harbor, ME). This species was chosen to match the species used from the source data used for drug prediction from Dohi et al [17], though this species is known to be more resistant to the development of TNBS colitis as compared to others [25,26]. The mice were divided evenly into drug treatment and control groups. TNBS 5% w/v in methanol was purchased from Fisher Scientific Company, LLC (Hampton, NH). TNBS at a dosage of 100mg/kg in 50% ethanol in a volume of 150μL was administered intrarectally to each mouse on day 0. For control mice, 150μL of 50% ethanol solution was used. Amrinone was purchased from Sigma-Aldrich, Inc (St. Louis, MO) and was administered at 10mg/kg in 1% v/v DMSO with saline at a dilution of 1mg/mL via intraperitoneal injection. Control mice received a 10mg/kg injection of saline. The treatment injections were administered daily from day 1 to day 4.

Mice were sacrificed on day 4, which was chosen to further match the experiments from the source data in Dohi et al [17], where mice were sacrificed on day 4. The colon was then dissected and harvested from each mouse. The tissue was fixed in formalin and paraffin-embedded. A longitudinal cross section slice of the colon was mounted on a slide and stained with hematoxylin and eosin.

Each slide was assessed in a blinded and randomized fashion by an independent veterinary pathologist. Each slide was graded based on ulceration, inflammation, edema, and fibroplasia. Ulceration, edema, and fibroplasia were considered as present or absent. Inflammation was graded as follows: 0 = no inflammation; 1 = small, focal areas limited to the lamina propria; 2 = multifocal or coalescing areas extending into the submucosa; 3 = transmural.

Given the heterogeneous and patchy nature of TNBS colitis, we assessed for the presence of fibroplasia, or wound healing, while taking into account the degree of induced colitis. Using R 3.6.1, we constructed a logistic regression model to assess the presence of fibroplasia. We used the degree of inflammation, the presence of ulceration, the presence of edema, and the treatment group as the covariates in our model.

## Results

### Integrating the connectivity map, drugbank, and reactome

Our network consists of 6,982 genes, that corresponds to the intersection of genes measured in CMap and genes present in Reactome. From DrugBank and CMap, we curated 453 drugs, which corresponded to 3,400 instances from CMap, including controls. These 453 drugs targeted 496 genes, with a median of 4 gene targets per drug.

### Similar drug mechanisms cluster together

**Fig 2:**
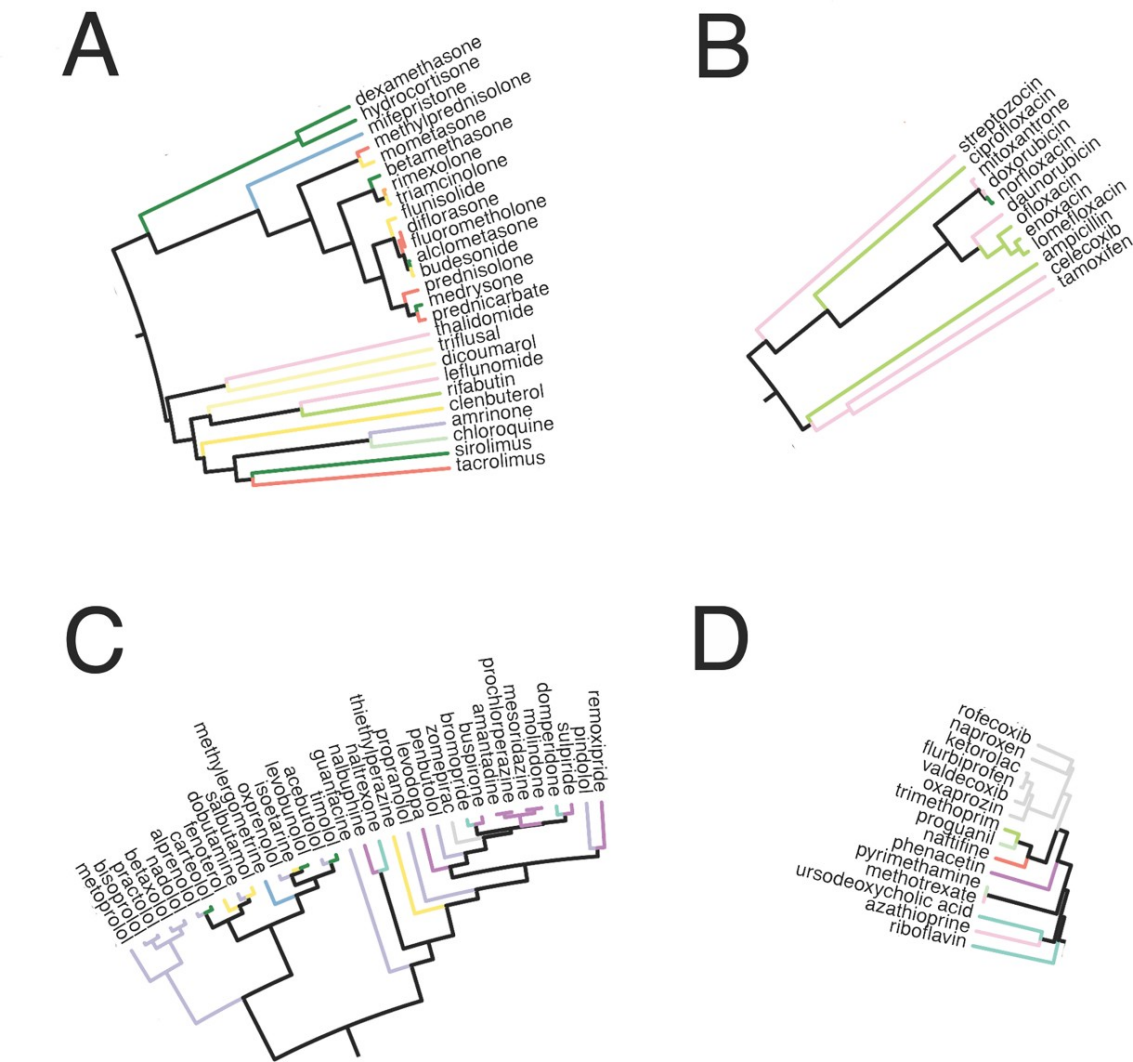
Excerpts from dendrogram shown in S1 Fig. Clusters of drugs emerge which share similar mechanisms, such as steroids and immunosuppressants (A), topoisomerase blockers used as antibiotics and chemotherapy (B), adrenergic and dopamine receptor drugs (C), and anti-inflammatory and immunomodulating drugs (D). Dendrogram branches are colored by the first level of the anatomic therapeutic chemical classification system (see S1 Fig for legend).

We first assessed the effects of the drugs on individual patients, to examine if similar drugs result in similar effects. As an example, using a drugged IBD sample derived from the first CD sample (GSM244753) in GSE9686, we clustered the drugged profiles (S1 Fig). The dendrogram reveals that drugs with similar mechanisms of action cluster together. Starting from the right, we see that the steroids cluster together, which includes topical and systemic steroids (Fig 2A). The neighborhood of drugs clustered near the steroids include additional anti-inflammatory drugs and immunosuppressants. For example, the cluster right below the steroids includes leflunomide, chloroquine, sirolimus, and tacrolimus (Fig 2A). Amrinone is present in this cluster, with chloroquine as its nearest neighbor. Though the steroids belong to four different categories of ATC codes due to being used in different disease processes, they all have a similar mechanism of action.

As we move counterclockwise, we come across a group of antibiotics mixed with chemotherapeutic agents (Fig 2B). These drugs block various forms of topoisomerase, with the antibiotics blocking bacterial topoisomerase and the chemotherapeutic agents blocking human topoisomerase.

Continuing along, the next large cluster along the top contains drugs that act on various receptors within the body, such as beta-adrenergic and dopamine receptors (Fig 2C). On the left, we see another section of anti-inflammatory drugs, including cyclooxygenase inhibitors and immunomodulatory drugs such as azathioprine (Fig 2D). The bottom portion of the circle contains drugs that are used for neuropsychiatric diseases, liver, and kidney issues, such as antipsychotics, diabetes and cholesterol medications, and diuretics (S1 Fig).

### Assessing drug response in GSE16879

**Fig 3:**
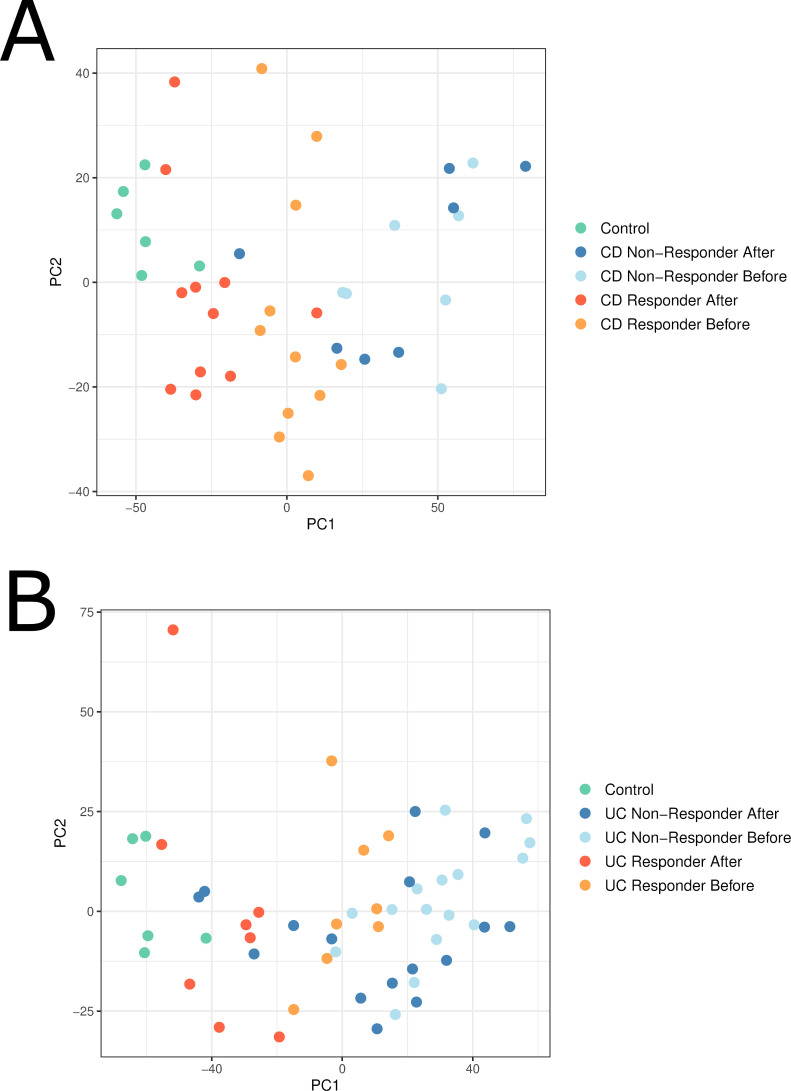
Principal components analysis showing drug responders and nonresponders before and after infliximab treatment in patients with CD (A) and UC (B) from GSE16879. Responder samples after treatment appear to migrate towards the control healthy samples.

Using the samples from GSE16879, we visualized treatment responders, treatment nonresponders, and controls for CD (Fig 3A) and UC patients (Fig 3B). Before treatment, responders and nonresponders tend to be located in close proximity in both CD and UC. For treatment nonresponders, there was minimal movement of the samples after treatment. For treatment responders, there is a shift of the after-response samples toward the cluster of healthy control samples. Overall, treatment responders after treatment were located significantly closer to healthy controls than treatment nonresponders for CD (p < 0.001) and for UC (p < 0.001). This indicates that the treatment appears to have an effect on the tissue that brings the sample closer to the healthy tissue, in part reversing some of the effects caused by the disease. The treated samples ultimately appear to exhibit an expression profile partway between the original disease state and healthy state. Our approach leverages this when creating our ranked drug list, comparing simulated drugged samples to healthy control samples.

### Comparing measured versus predicted methotrexate response

**Fig 4:**
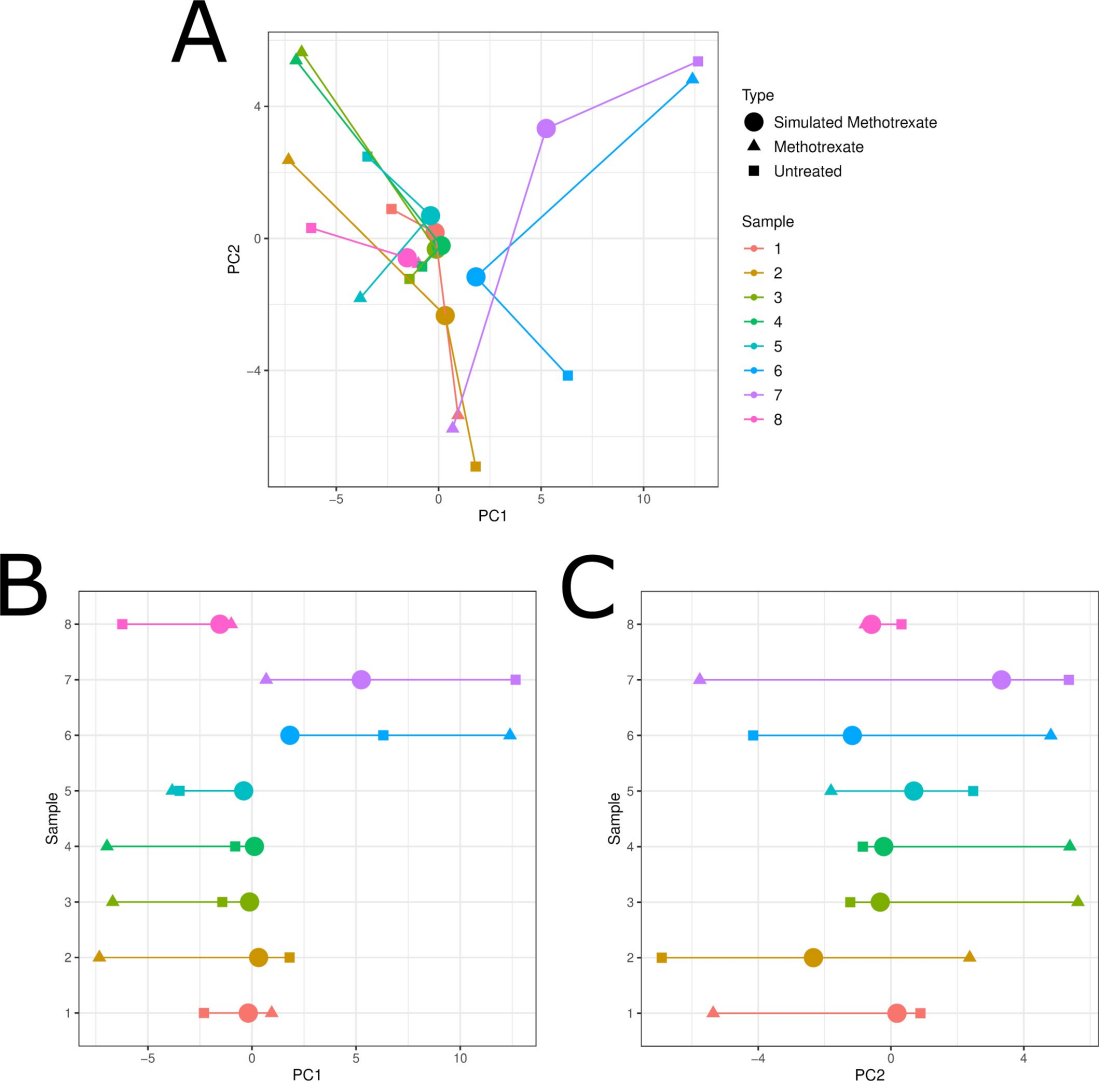
(A) PCA showing untreated, methotrexate treated, and simulated methotrexate treated samples generated by NetPTP for samples in GSE45867. Samples were further visualized along PC1 (B) and PC2 (C) separately, showing that simulated methotrexate samples are located between untreated and measured methotrexate samples along PC2. Our method seems conservative in that the simulated treatment samples tend to remain closer to the untreated samples as compared to the treated samples.

We visualized our simulated methotrexate treatment samples versus untreated and actual methotrexate treatment samples using principal components analysis (Fig 4). Notably, untreated samples and methotrexate treated samples did not cluster together but were scattered across both principal component 1 (PC1) and principal component 2 (PC2) (Fig 4A). PC1 and PC2 accounted for 29.1% and 13.2% of the variance respectively. The simulated methotrexate samples were generally located on the trajectory between the untreated and treated samples, with some deviance towards the center of the plot. When analyzing PC1 and PC2 separately (Fig 4B and 4C), the simulated methotrexate samples always fell in between the untreated and actual treated samples for PC2 (Fig 4C).

### Drug rankings in human IBD samples

We applied NetPTP to four publicly available IBD datasets: GSE9686 (11 CD, 5 UC, 8 healthy) [13], GSE16879 (19 CD, 24 UC, 6 healthy) [12], GSE10616 (14 CD, 10 UC, 11 healthy) [14], and GSE36807 (13 CD, 15 UC, 7 healthy) [15]. The top 10 ranked drugs per study for CD are shown in Table 1 and for UC are shown in Table 2. Averaging the rankings across all patients in GSE9686, GSE10616, and GSE36807 yielded the top drugs shown in Table 3.

**Table 1:** Average drug rankings for each GEO study for CD samples.

Rank	GSE9686	GSE10616	GSE36807	GSE16879
1	furosemide	furosemide	propofol	mesalazine
2	ribostamycin	hydrochlorothiazide	primidone	hydrocortisone
3	ivermectin	bendroflumethiazide	colforsin	sulfasalazine
4	metolazone	ivermectin	valproic acid	methazolamide
5	piretanide	piretanide	selegiline	rimexolone
6	puromycin	thiocolchicoside	procainamide	triamcinolone
7	thiocolchicoside	quinethazone	biotin	betamethasone
8	torasemide	torasemide	etodolac	medrysone
9	hydrochlorothiazide	metolazone	bupropion	tenoxicam
10	hydrocortisone	benzthiazide	mebendazole	nabumetone

**Table 2:** Average drug rankings for each GEO study for UC samples.

Rank	GSE9686	GSE10616	GSE36807	GSE16879
1	dextromethorphan	cefoxtaxime	prednisolone	thichlormethiazide
2	prednisone	dextromethorphan	alclometasone	diazoxide
3	sulfinpyrazone	methazolamide	budesonide	methazoldamide
4	cefotaxime	brinzolamide	hydrocortisone	hydroflumethiazide
5	rimexolone	streptozocin	fluorometholone	furosemide
6	triamcinolone	biotin	prednicarbate	hydrochlorothiazide
7	betamethasone	diclofenamide	diflorasone	hydrocortisone
8	doxazosin	triamcinolone	flunisolide	astemizole
9	alfuzosin	rimexolone	medrysone	bendroflumethiazide
10	medrysone	betamethasone	betamethasone	terfenadine

**Table 3:** Overall average drug rankings for CD and UC human colonic samples.

Rank	CD	UC
1	furosemide	diazoxide
2	propofol	methazolamide
3	metolazone	hydrocortisone
4	hydrochlorothiazide	trichlormethiazide
5	ivermectin	hydroflumethiazide
6	methazolamide	brinzolamide
7	ribostamycin	furosemide
8	thiocolchicoside	hydrochlorothiazide
9	piretanide	diclofenamide
10	mesalazine	rimexolone

GSE16879 consists of patients that are refractory to corticosteroids and/or immunosuppression, with 7 UC patients and 6 CD patients on corticosteroids at baseline, per Table 1 of Arijs et al [12]. We evaluated the rank of prednisone in GSE16879 versus the other studies. For patients in GSE16879, the rank of prednisone was significantly higher than the patients from the other 3 studies (p = 0.028), indicating prednisone was predicted to be less effective in patients in GSE16879. The median in GSE16879 was 261 with an interquartile range (IQR) of 154.5–338.5 versus a median of 178 and an IQR of 68.25–297.25 in the other patients.

**Fig 5:**
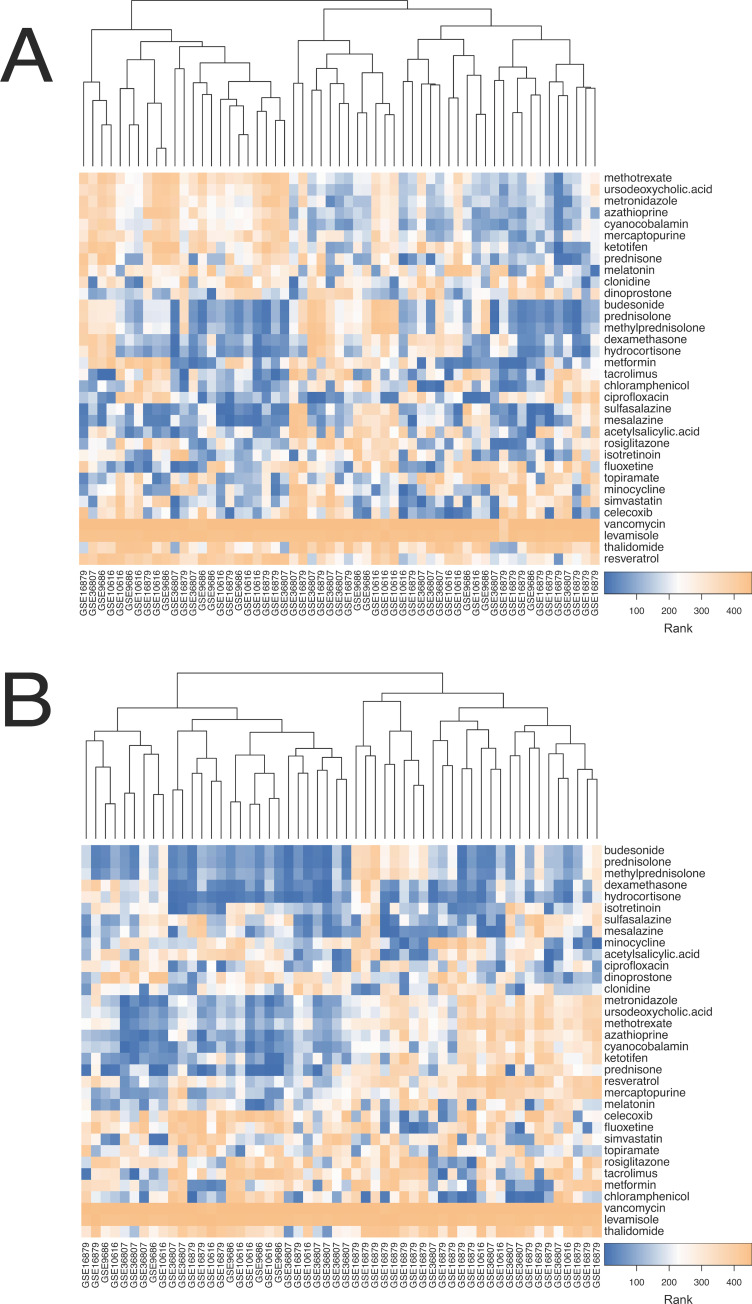
Heatmaps showing drug ranks for IBD related drugs for patients with CD (A) and UC (B) from GSE16879, GSE10616, GSE36807, and GSE9686. Blue indicates a drug with higher predicted efficacy and orange indicates one with lower predicted efficacy.

We next assessed the rankings of drugs previously associated with IBD in the literature for CD (Fig 5A) and UC samples (Fig 5B). In CD patients (Fig 5A), three different subgroups of patients appear, and samples do not cluster by study. Patients on the left are predicted to have good response to anti-inflammatory medication and steroids, but not to immunomodulators such as azathioprine. Those in the middle show the opposite pattern, with good predicted response to immunomodulators but not anti-inflammatory medications, and those on the right tend to have high ranks for all classes.

In UC patients (Fig 5B), we again see that samples do not cluster by study, but instead in two main groups. Patients on the left exhibit a high ranking for steroids, anti-inflammatory medications, and immunomodulators. In contrast, patients on the right are predicted to have poor efficacy to immunomodulators but maintain favorable rankings for steroids and anti-inflammatory medications.

### Comparison to drug-disease score rankings

We compared the ranking of nine known IBD treatments generated by NetPTP against the rankings generated by the DDS from the method in Sirota and Dudley et al [23]. Rankings were generated for each drug-disease pair for 9 drugs, 2 diseases (CD, UC), and in 3 studies, resulting in N = 54. Using the Wilcoxon rank-sum paired test, our NetPTP rankings of the known IBD treatments were significantly more favorable than the DDS rankings (p = 0.0011). Overall, where a lower ranking is more favorable, our rankings of known IBD treatments had a median ranking of 102.5 (interquartile range 46.5–205.2) and the DDS rankings had a median of 243.5 (interquartile range 128.2–331.8).

### Drug rankings in mouse IBD samples

**Fig 6:**
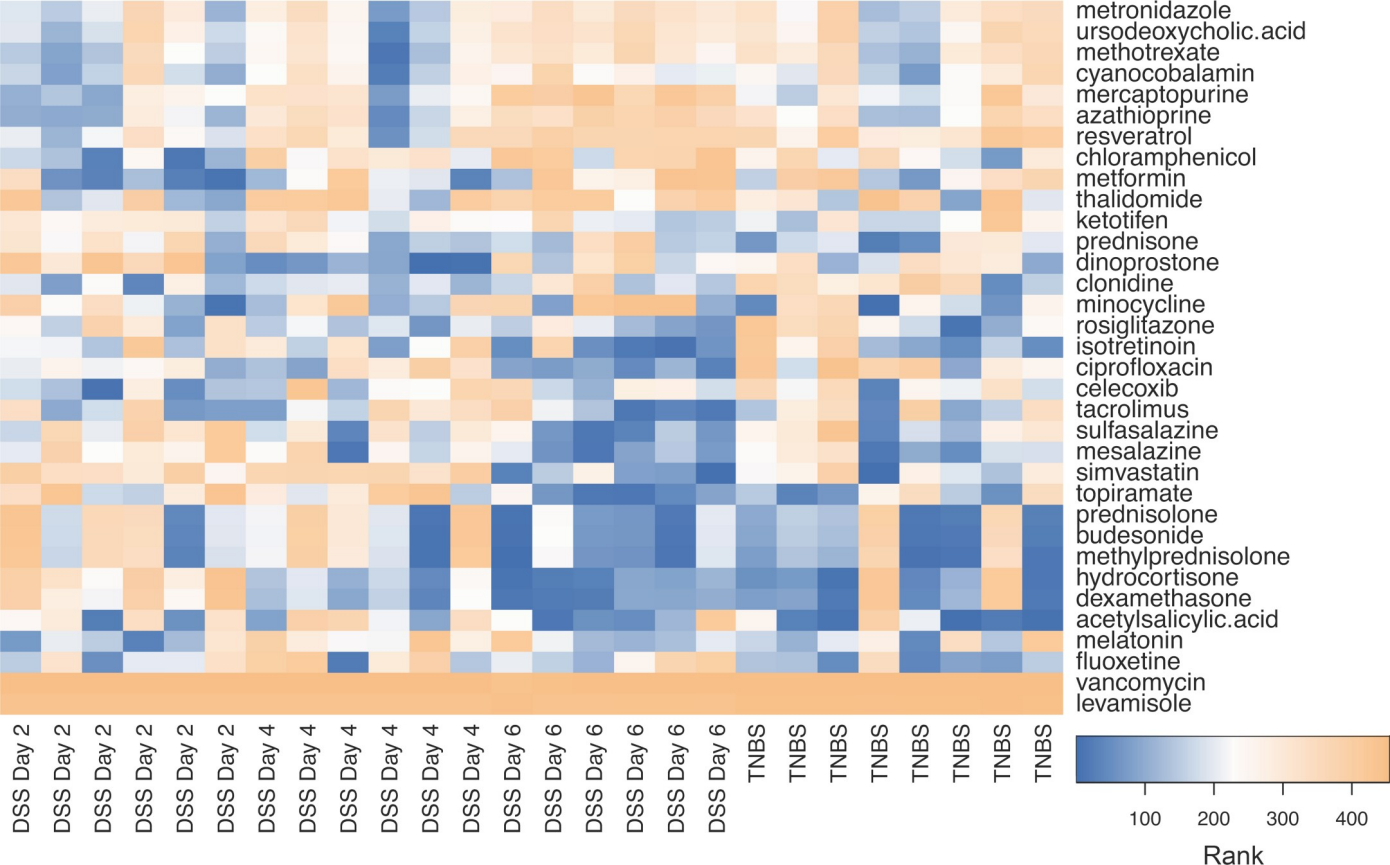
Heatmap showing drug ranks for IBD related drugs for two mouse IBD colonic sample studies from GEO. Blue indicates a drug with higher predicted efficacy and orange indicates one with lower predicted efficacy.

**Fig 7:**
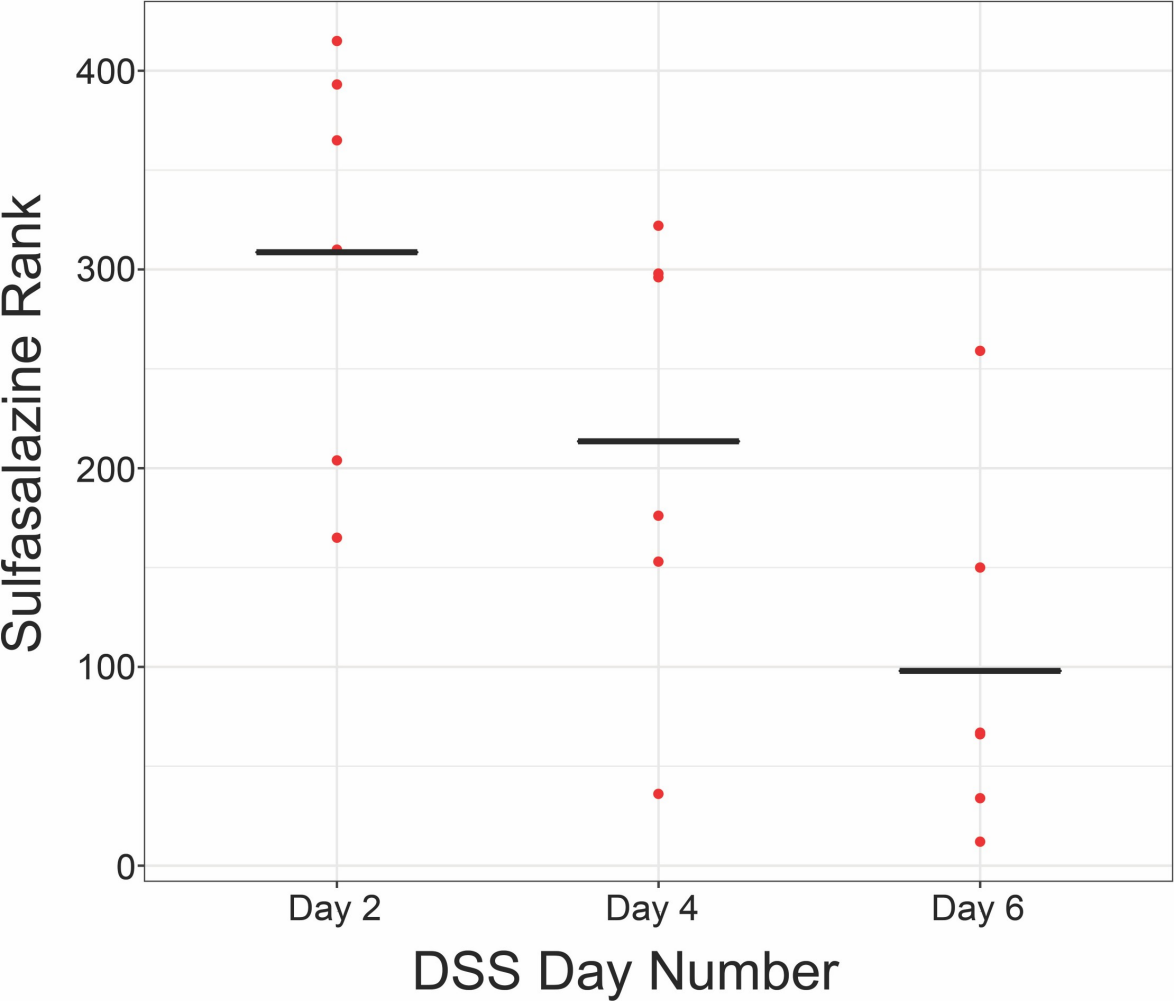
Sulfasalazine rank significantly decreases (p = 0.01) from day 2 to day 6 of DSS administration for mice colonic samples from GSE22307. The mean is indicated by the black bars.

Fig 6 depicts the rankings of previous IBD drugs in literature for the DSS day 2, day 4, day 6, and TNBS samples from GSE22307 [16] and GSE53835 [17]. As we progress from day 2 to day 6 of DSS administration and the inflammation is being induced, the drug ranking pattern changes from immunomodulators being highly ranked to ant-inflammatory drugs and steroids being highly ranked. For example, sulfasalazine significantly improves in rank as the inflammation progresses (Fig 7, p = 0.01). By the time we reach day 6, the pattern generally resembles the TNBS samples, which represent acute inflammation of the colon. For day 6 DSS mice and TNBS mice, the top 10 drugs are shown in Table 4.

**Table 4:** Overall average drug rankings for DSS and TNBS mouse colonic samples.

Rank	DSS Day 2	DSS Day 4	DSS Day 6	TNBS
1	hydralazine	mepyramine	hydrochlorothiazide	amrinone
2	probenecid	chlorcyclizine	bendroflumethiazide	chloroquine
3	gliclazide	diphenhydramine	ivermectin	puromycin
4	maprotiline	alimemazine	bumetanide	etomidate
5	vinblastine	menhydrinate	etomidate	ivermectin
6	cAMP	triprolidine	gliquidone	flucytosine
7	phenformin	clemastine	quinethazone	azacytidine
8	probucol	cyclizine	flucinonide	triflusal
9	metformin	cypropheptadine	metolazone	ribostamycin
10	milrinone	cetirizine	furosemide	decitabine

### Experimental evaluation of top ranked DRUG in TNBS mice

We constructed a logistic regression model to assess the presence of wound healing in TNBS mice given our top prediction, amrinone, versus saline controls (Table 5). Drug status was treated as 1 for amrinone, and 0 for saline. A higher level of inflammation or the presence of ulceration led to a greater likelihood of the presence of recovery, whereas edema had the opposite effect. Those mice that were in the amrinone treatment group had a coefficient of 2.06, corresponding to an odds ratio of 7.75.

**Table 5:** Logistic regression model assessing fibroplasia in TNBS mice.

Variable	Log Odds Ratio	95% Confidence Interval	p-value
Drug Status	2.05	(-0.82, 5.71)	0.180
Inflammation	0.86	(-0.78, 3.06)	0.340
Edema	-0.74	(-4.57, 2.13)	0.651
Ulceration	2.14	(-0.91, 6.77)	0.237

## Discussion

In this work, we present NetPTP, a novel systems pharmacological approach for drug repurposing and modeling drug effects. In our approach, we model drug effects and apply these effects to diseased samples to assess which samples exhibit the closest return to a healthy profile, thus leading to individualized predictions of drug efficacy. NetPTP was motivated in part by previous studies [6] and our analysis of the samples in GSE16879, which showed that infliximab responder samples were located significantly closer to healthy controls than non-responder samples. By assessing for a shift toward healthy controls, we believe we may not only predict the efficacy of the drug in treating the disease but may also rank drugs higher that have fewer adverse effects.

We assessed NetPTP using methotrexate treated samples from GSE45867, which studied another autoimmune disease, rheumatoid arthritis. Our simulated drugged samples were generally located on the trajectory between the untreated and the treated samples. NetPTP appears to capture the direction of the effect of the drug and then subsequently apply the learned effect to new data samples, producing simulated drugged samples which represent a conservative step from the untreated sample towards the treated sample. In particular, the model’s prediction fell between the untreated and treated sample for all eight samples along principal component 2. Thus, this particular principal component may reflect more of the biological changes induced with methotrexate treatment. Delving deeper, the gene with the largest absolute weight in PC2 is PMAIP1, also known as NOXA. NOXA plays a role in the apoptosis pathway as part of the BCL-2 family and is regulated by p53 [27]. Methotrexate has been shown to mediate apoptosis via upregulation of p53 and its downstream targets, including NOXA [28]. NOXA has been shown to induce apoptosis of fibroblast-like synoviocytes [29] and bone osteoclasts [30], both of which are thought to have a role in the pathogenesis of rheumatoid arthritis [31].

We applied NetPTP to multiple publicly available CD and UC datasets with human colonic samples. Clustering the simulated drugged profiles for one patient revealed that drugs with similar mechanisms cluster together (Fig 2). When clustering our patients and their rankings with IBD drugs in literature, we found that patients grouped into multiple different treatment response profiles (Fig 5). This suggests that it may be possible to guide treatment decision making, particularly when deciding between multiple first line treatment options.

In CD samples, we see that of the ten top ranked drugs, mesalazine, a known IBD drug, is included (Table 3). The others are diuretics, antimicrobials, and two GABA antagonists, including thiocolchicoside which has anti-inflammatory effects [32]. This list suggests that these CD samples tend to have a fluid overloaded state, likely due to edema, with some dysregulation of the enteric nervous system and inflammation. In UC, though we have some of the same diuretic medications, we also see steroids more often, for example as the top ranked drug in GSE36807 (Table 2, Table 3). The top overall ranked drug, diazoxide, is a potassium channel activator, and has been shown to heal acute gastric ulcers in rats [33]. Carbonic anhydrase inhibitors are also present on both top ranked lists, and previous studies have shown targeting carbonic anhydrase I and IV have ameliorated IBD in mouse models [34,35].

We compared the rankings generated by NetPTP to the drug-disease score (DDS) generated by Sirota and Dudley et al [23]. Overall, our method produced significantly better rankings for the nine known IBD drugs in the Connectivity Map. However, as IBD is a heterogenous disease, some of the study patients may have not been responsive to each and every one of these therapies. We compared our results at the study level, as calculating the DDS uses SAM, which aggregates multiple patients to derive up-regulated and down-regulated genes. NetPTP aims to address some of these limitations by providing predictions at the patient level while incorporating the connections between genes to capture more of the underlying biology driving drug response.

In mouse samples, we analyzed the drug rankings over the course of acute inflammation development in DSS mice and in TNBS mice. We found that as inflammation progressed from day 2 to day 6, known treatments such as sulfasalazine became significantly more highly ranked (Fig 7), and the day 6 DSS drug rankings became more similar to the drug rankings seen with the TNBS mice (Fig 6). The top drug ranked in the TNBS mice was amrinone, a phosphodiesterase (PDE) type 3 inhibitor with some effect on PDE type 4, which was clustered with other immunosuppressants (Fig 2A). Phosphodiesterases control the concentration of cyclic adenosine monophosphate, which suppresses inflammation via the NF-κB pathway. Amrinone has long been shown to have anti-inflammatory effects [36,37], and PDE4 inhibitors have recently been suggested as a new avenue for IBD drugs [38].

We assessed this top drug, amrinone, in a preliminary study using a TNBS mouse model, using the same mouse strain as used in GSE53835. We assessed the presence of fibroplasia in twenty mice, taking into account the degree of inflammation and the presence of ulceration, as this particular strain is moderately resistant to TNBS and to account for the heterogenous nature of the TNBS model [25,26]. We evaluated the drug effect on day 4 in keeping with the source data; however, these experiments may benefit from a longer time course to better characterize the drug effect. Though the drug status did not reach statistical significance, it appears to be one of the more important covariates for predicting the presence of fibroplasia in these mice and would merit further investigation in a larger study.

Our approach involves curating and combining multiple publicly available resources, including Reactome [20], the Connectivity Map [39], DrugBank [19], and the Gene Expression Omnibus. Though CMap includes hundreds of drugs, some IBD therapies, such as infliximab, are not represented. Furthermore, transcriptomics data are shifting from gene expression to RNA-seq. For IBD, currently most published studies for public use are gene expression data. However, our approach can easily translate to RNA-seq data and be expanded to include more drugs, such as data from the Library of Integrated Network-Based Cellular Signatures (LINCS) project (http://www.lincsproject.org/).

In addition to different sources of drugged data, the other aspects of NetPTP are also modular and can be easily adjusted to accommodate different network architectures, network data from different species, additional drugs and drug targets, and additional diseases of interest. As NetPTP can use different sources of healthy control data, it could also be used to rank drugs based on a patient’s own healthy samples before he or she developed a disease. In addition to being able to incorporate different sources of transcriptomic data, NetPTP works with any pre-defined set of edges that has been converted to a directed network, such as Reactome, the Kyoto Encyclopedia of Genes and Genomes [40], the Search Tool for the Retrieval of Interacting Genes/Proteins (STRING) [41], or networks derived from experimental data. With time, we hope the increasing amount of publicly available data available and a wider selection of drugged data including newer therapies such as monoclonal antibodies will expand the applicability and utility of our method, offering personalized treatment regimens as well as identifying novel treatment avenues for IBD.

## Supporting information

S1 Fig Dendrogram of drugged profiles of a CD patient from GSE9686. Dendrogram branches are colored by the first level of the anatomic therapeutic chemical classification system.
(TIF)

S1 Table Dataset summary for publicly available mouse and human data. (DOCX)

## References

[1] Wouters OJ, McKee M, Luyten J. Estimated Research and Development Investment Needed to Bring a New Medicine to Market, 2009–2018. JAMA—J Am Med Assoc. 2020;323: 844–853. 10.1001/jama.2020.1166 32125404 PMC7054832

[2] Li L, Greene I, Readhead B, Menon MC, Kidd BA, Uzilov A V., et al Novel therapeutics identification for fibrosis in renal allograft using integrative informatics approach. Sci Rep. 2017;7: 1–14. 10.1038/s41598-016-0028-x 28051114 PMC5209709

[3] Van Noort V, Schölch S, Iskar M, Zeller G, Ostertag K, Schweitzer C, et al Novel drug candidates for the treatment of metastatic colorectal cancer through global inverse gene-expression profiling. Cancer Res. 2014;74: 5690–5699. 10.1158/0008-5472.CAN-13-3540 25038229

[4] Ananthakrishnan AN. Epidemiology and risk factors for IBD. Nat Rev Gastroenterol Hepatol. 2015;12: 205–217. 10.1038/nrgastro.2015.34 25732745

[5] Neurath MF. Current and emerging therapeutic targets for IBD. Nat Rev Gastroenterol Hepatol. 2017;14: 269–278. 10.1038/nrgastro.2016.208 28144028

[6] Dudley JT, Sirota M, Shenoy M, Pai RK, Roedder S, Chiang AP, et al Computational repositioning of the anticonvulsant topiramate for inflammatory bowel disease. Sci Transl Med. 2011;3: 96ra76 10.1126/scitranslmed.3002648 21849664 PMC3479650

[7] Collij V, Festen EAM, Alberts R, Weersma RK. Drug Repositioning in Inflammatory Bowel Disease Based on Genetic Information. Inflamm Bowel Dis. 2016;22: 2562–2570. 10.1097/MIB.0000000000000912 27753694

[8] Grenier L, Hu P. Computational drug repurposing for inflammatory bowel disease using genetic information. Comput Struct Biotechnol J. 2019;17: 127–135. 10.1016/j.csbj.2019.01.001 30728920 PMC6352300

[9] Irizarry R a Bolstad BM, Collin F Cope LM, Hobbs B Speed TP. Summaries of Affymetrix GeneChip probe level data. Nucleic Acids Res. 2003;31: e15 10.1093/nar/gng015 12582260 PMC150247

[10] Johnson WE, Li C, Rabinovic A. Adjusting batch effects in microarray expression data using empirical Bayes methods. Biostatistics. 2007;8: 118–127. 10.1093/biostatistics/kxj037 16632515

[11] Leek JT, Johnson WE, Parker HS, Fertif EJ, Jaffe AE, Storey JD. sva: Surrogate Variable Analysis. R package version 3.20.0. 2016.

[12] Arijs I, De Hertogh G, Lemaire K, Quintens R, Van Lommel L, Van Steen K, et al Mucosal gene expression of antimicrobial peptides in inflammatory bowel disease before and after first infliximab treatment. PLoS One. 2009;4: e7984 10.1371/journal.pone.0007984 19956723 PMC2776509

[13] Carey R, Jurickova I, Ballard E, Bonkowski E, Han X, Xu H, et al Activation of an IL-6:STAT3-dependent transcriptome in pediatric-onset inflammatory bowel disease. Inflamm Bowel Dis. 2008;14: 446–457. 10.1002/ibd.20342 18069684 PMC2581837

[14] Kugathasan S, Baldassano RN, Bradfield JP, Sleiman PMA, Imielinski M, Guthery SL, et al Loci on 20q13 and 21q22 are associated with pediatric-onset inflammatory bowel disease. Nat Genet. 2008;40: 1211–1215. 10.1038/ng.203 18758464 PMC2770437

[15] Montero-Meléndez T, Llor X, García-Planella E, Perretti M, Suárez A. Identification of Novel Predictor Classifiers for Inflammatory Bowel Disease by Gene Expression Profiling. Calogero RA, editor. PLoS One. 2013;8: e76235 10.1371/journal.pone.0076235 24155895 PMC3796518

[16] Fang K, Bruce M, Pattillo CB, Zhang S, Stone R, Clifford J, et al Temporal genomewide expression profiling of DSS colitis reveals novel inflammatory and angiogenesis genes similar to ulcerative colitis. Physiol Genomics. 2011;43: 43–56. 10.1152/physiolgenomics.00138.2010 20923862 PMC3026350

[17] Dohi T, Kawashima R, Kawamura YI, Otsubo T, Hagiwara T, Amatucci A, et al Pathological activation of canonical nuclear-factor κB by synergy of tumor necrosis factor α and TNF-like weak inducer of apoptosis in mouse acute colitis. Cytokine. 2014;69: 14–21. 10.1016/j.cyto.2014.05.001 25022957

[18] Ducreux J, Durez P, Galant C, Toukap AN, Van Den Eynde B, Houssiau FA, et al Global molecular effects of tocilizumab therapy in rheumatoid arthritis synovium. Arthritis Rheumatol. 2014;66: 15–23. 10.1002/art.38202 24449571

[19] Wishart DS, Knox C, Guo AC, Shrivastava S, Hassanali M, Stothard P, et al DrugBank: a comprehensive resource for in silico drug discovery and exploration. Nucleic Acids Res. 2006;34: D668–D672. Available: http://eutils.ncbi.nlm.nih.gov/entrez/eutils/elink.fcgi?dbfrom=pubmed&id=16381955&retmode=ref&cmd=prlinks. 10.1093/nar/gkj067 16381955 PMC1347430

[20] Croft D, O’Kelly G, Wu G, Haw R, Gillespie M, Matthews L, et al Reactome: A database of reactions, pathways and biological processes. Nucleic Acids Res. 2011;39: 691–697. 10.1093/nar/gkq1018 21067998 PMC3013646

[21] Percha B, Altman RB. Learning the Structure of Biomedical Relationships from Unstructured Text. PLoS Comput Biol. 2015;11: 1–27. 10.1371/journal.pcbi.1004216 26219079 PMC4517797

[22] Percha B, Altman RB. A global network of biomedical relationships derived from text. Bioinformatics. 2018;34: 2614–2624. 10.1093/bioinformatics/bty114 29490008 PMC6061699

[23] Sirota M, Dudley JT, Kim J, Chiang AP, Morgan AA, Sweet-Cordero A, et al Discovery and Preclinical Validation of Drug Indications Using Compendia of Public Gene Expression Data. Sci Transl Med. 2011;3: 96ra77–96ra77. 10.1126/scitranslmed.3001318 21849665 PMC3502016

[24] Tusher VG, Tibshirani R, Chu G. Significance analysis of microarrays applied to the ionizing radiation response. Proc Natl Acad Sci. 2001;98: 5116–5121. 10.1073/pnas.091062498 11309499 PMC33173

[25] Scheiffele F, Fuss IJ. Induction of TNBS Colitis in Mice. Curr Protoc Immunol. 2002;Chapter 15: 1–14. 10.1002/0471142735.im1519s49 18432874

[26] Te Velde AA, Verstege MI, Hommes DW. Critical appraisal of the current practice in murine TNBS-induced colitis. Inflamm Bowel Dis. 2006;12: 995–999. 10.1097/01.mib.0000227817.54969.5e 17012970

[27] Ploner C, Kofler R, Villunger A. Noxa: at the tip of the balance between life and death. Oncogene. 2008;27 Suppl 1: S84–92. 10.1038/onc.2009.46 19641509 PMC3272398

[28] Huang WY, Yang PM, Chang YF, Marquez VE, Chen CC. Methotrexate induces apoptosis through p53/p21-dependent pathway and increases E-cadherin expression through downregulation of HDAC/EZH2. Biochem Pharmacol. 2011;81: 510–517. 10.1016/j.bcp.2010.11.014 21114963

[29] Leech M, Lacey D, Xue JR, Santos L, Hutchinson P, Wolvetang E, et al Regulation of p53 by macrophage migration inhibitory factor in inflammatory arthritis. Arthritis Rheum. 2003;48: 1881–1889. 10.1002/art.11165 12847682

[30] Idrus E, Nakashima T, Wang L, Hayashi M, Okamoto K, Kodama T, et al The role of the BH3-only protein Noxa in bone homeostasis. Biochem Biophys Res Commun. 2011;410: 620–625. 10.1016/j.bbrc.2011.06.040 21689638

[31] Pintao MC, Ribeiro DD, Bezemer ID, Garcia AA, de Visser MCH, Doggen CJM, et al Protein S levels and the risk of venous thrombosis: results from the MEGA case-control study. Blood. 2013;122: 3210–3219. Available: http://www.bloodjournal.org/cgi/doi/10.1182/blood-2013-04-499335. 24014240 10.1182/blood-2013-04-499335

[32] Reuter S, Prasad S, Phromnoi K, Ravindran J, Sung B, Yadav VR, et al Thiocolchicoside exhibits anticancer effects through downregulation of NF-κB pathway and its regulated gene products linked to inflammation and cancer. Cancer Prev Res. 2010;3: 1462–1472. 10.1158/1940-6207.CAPR-10-0037 20978115 PMC3142676

[33] Rahgozar M, Pazokitoroudi H, Bakhtiarian A, Djahanguiri B. Diazoxide, a KATP opener, accelerates restitution of ethanol or indomethacin-induced gastric ulceration in rats independent of polyamines. J Gastroenterol Hepatol. 2001;16: 290–296. 10.1046/j.1440-1746.2001.02433.x 11339420

[34] Yamanishi H, Murakami H, Ikeda Y, Abe M, Kumagi T, Hiasa Y, et al Regulatory Dendritic Cells Pulsed with Carbonic Anhydrase I Protect Mice from Colitis Induced by CD4+CD25- T Cells. J Immunol. 2012;188: 2164–2172. 10.4049/jimmunol.1100559 22291189

[35] Mizoguchi E, Xavier RJ, Reinecker HC, Uchino H, Bhan AK, Podolsky DK, et al Colonic epithelial functional phenotype varies with type and phase of experimental colitis. Gastroenterology. 2003;125: 148–161. 10.1016/s0016-5085(03)00665-6 12851880

[36] Németh ZH, Szabó C, Haskó G, Salzman AL, Vizi ES. Effect of the phosphodiesterase III inhibitor amrinone on cytokine and nitric oxide production in immunostimulated J774.1 macrophages. Eur J Pharmacol. 1997;339: 215–221. 10.1016/s0014-2999(97)01392-7 9473138

[37] Takeuchi K, del Nido PJ, Ibrahim AE, Cao-Danh H, Friehs I, Glynn P, et al Vesnarinone and amrinone reduce the systemic inflammatory response syndrome. J Thorac Cardiovasc Surg. 1999;117: 375–382. 10.1016/S0022-5223(99)70436-8 9918980

[38] Spadaccini M, D’Alessio S, Peyrin-Biroulet L, Danese S. PDE4 inhibition and inflammatory bowel disease: A novel therapeutic avenue. Int J Mol Sci. 2017;18: 1–14. 10.3390/ijms18061276 28617319 PMC5486098

[39] Lamb J. The Connectivity Map: a new tool for biomedical research. Nat Rev Cancer. 2007;7: 54–60. 10.1038/nrc2044 17186018

[40] Kanehisa M, Goto S. KEGG: kyoto encyclopedia of genes and genomes. Nucleic Acids Res. 2000;28: 27–30. Available: http://www.ncbi.nlm.nih.gov/pubmed/10592173. 10.1093/nar/28.1.27 10592173 PMC102409

[41] von Mering C. STRING: known and predicted protein-protein associations, integrated and transferred across organisms. Nucleic Acids Res. 2004;33: D433–D437. 10.1093/nar/gki005 15608232 PMC539959

