# Supplementary figures and images for: Modeling drug response using network-based personalized treatment prediction (NetPTP) with applications to inflammatory bowel disease
Source: PLoS Comput Biol. 2021 Feb 5;17(2):e1008631. doi: 10.1371/journal.pcbi.1008631 (PMC7891788; doi:10.1371/journal.pcbi.1008631)

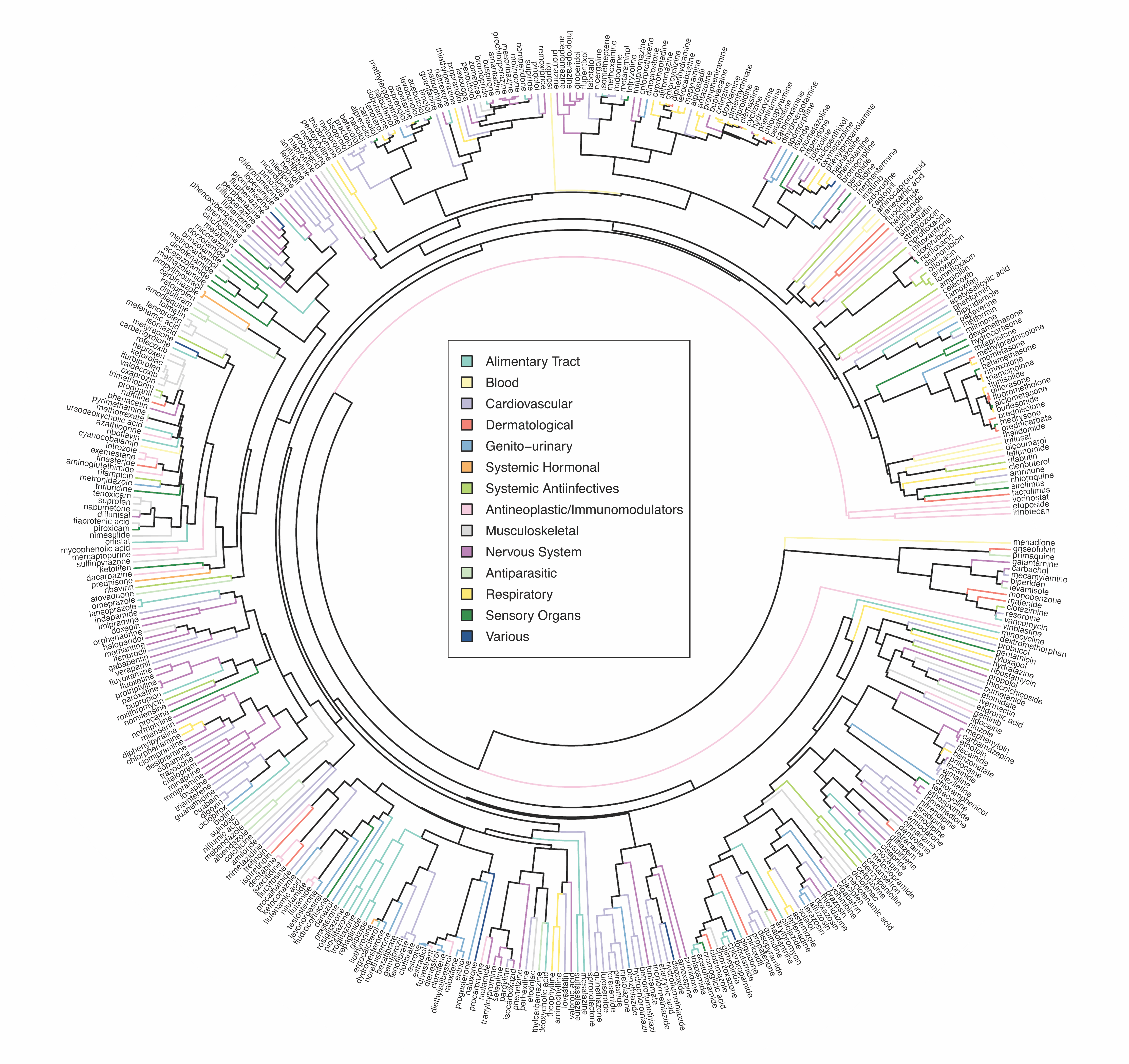

Supplement: S1 Fig — Dendrogram branches are colored by the first level of the anatomic therapeutic chemical classification system. (TIF) [file pcbi.1008631.s001.tif]
