# Supplementary material for: Modeling drug response using network-based personalized treatment prediction (NetPTP) with applications to inflammatory bowel disease
Source: PLoS Comput Biol. 2021 Feb 5;17(2):e1008631. doi: 10.1371/journal.pcbi.1008631 (PMC7891788; doi:10.1371/journal.pcbi.1008631)
Supplement: S1 Table — (DOCX) [file pcbi.1008631.s002.docx]

Table S1: Dataset summary for publicly available mouse and human data.

| Accession | Platform | Species | # Samples | Study Question | Reference |
| --- | --- | --- | --- | --- | --- |
| GSE9686 | Affymetrix U133 Plus 2.0 | Human | 33 | Analyzing expression of the IL-6:STAT3 pathway | Carey et al. 2008 [1] |
| GSE16879 | Affymetrix U133 Plus 2.0 | Human | 133 | Identifying genes predictive of infliximab response | Arijs et al. 2009 [2] |
| GSE10616 | Affymetrix U133 Plus 2.0 | Human | 58 | Characterizing colonic gene expression in IBD | Kugathasan et al. 2008 [3] |
| GSE36807 | Affymetrix U133 Plus 2.0 | Human | 35 | Distinguishing phenotypes of IBD | Montero-Melendez et al. 2013 [4] |
| GSE22307 | Affymetrix Mouse Genome 430 2.0 | Mouse | 23 | Investigating temporal gene expression changes in DSS mice | Fang et al. 2011[5] |
| GSE53835 | Affymetrix Mouse Genome 430 2.0 | Mouse | 72 | Testing TNFR-Fc and anti-TWEAK in TNBS mice | Dohi et al. 2014 [6] |

References

1. Carey R, Jurickova I, Ballard E, Bonkowski E, Han X, Xu H, et al. Activation of an IL-6:STAT3-dependent transcriptome in pediatric-onset inflammatory bowel disease. Inflamm Bowel Dis. 2008;14: 446–457. doi:10.1002/ibd.20342

2. Arijs I, De Hertogh G, Lemaire K, Quintens R, Van Lommel L, Van Steen K, et al. Mucosal gene expression of antimicrobial peptides in inflammatory bowel disease before and after first infliximab treatment. PLoS One. 2009;4: e7984. doi:10.1371/journal.pone.0007984

3. Kugathasan S, Baldassano RN, Bradfield JP, Sleiman PMA, Imielinski M, Guthery SL, et al. Loci on 20q13 and 21q22 are associated with pediatric-onset inflammatory bowel disease. Nat Genet. 2008;40: 1211–1215. doi:10.1038/ng.203

4. Montero-Meléndez T, Llor X, García-Planella E, Perretti M, Suárez A. Identification of Novel Predictor Classifiers for Inflammatory Bowel Disease by Gene Expression Profiling. Calogero RA, editor. PLoS One. 2013;8: e76235. doi:10.1371/journal.pone.0076235

5. Fang K, Bruce M, Pattillo CB, Zhang S, Stone R, Clifford J, et al. Temporal genomewide expression profiling of DSS colitis reveals novel inflammatory and angiogenesis genes similar to ulcerative colitis. Physiol Genomics. 2011;43: 43–56. doi:10.1152/physiolgenomics.00138.2010

6. Dohi T, Kawashima R, Kawamura YI, Otsubo T, Hagiwara T, Amatucci A, et al. Pathological activation of canonical nuclear-factor κB by synergy of tumor necrosis factor α and TNF-like weak inducer of apoptosis in mouse acute colitis. Cytokine. 2014;69: 14–21. doi:10.1016/j.cyto.2014.05.001
